# Anti-glaucoma potential of hesperidin in experimental glaucoma induced rats

**DOI:** 10.1186/s13568-020-01027-1

**Published:** 2020-05-18

**Authors:** Baiyang Lu, Xue Wang, Zengjin Ren, Haitao Jiang, Bingqian Liu

**Affiliations:** Department of Ophthalmology, The First Affiliated Hospital of Kangda College of Nanjing Medical University, Xuzhou Medical University Affiliated Hospital of Lianyungang, The First People’s Hospital of Lianyungang, No. 6 Zhenhua East Road, Haizhou District, Lianyungang, 222002 China

**Keywords:** Hesperidin, Glutathione, Glutamate, Hypertension, Rats

## Abstract

Glaucoma is well-known clinical eye conditions that damage the optic nerve due to abnormal pressure conditions in eye. Hesperidin is well-known glycoside widely present in the citrus fruits, and its aglycone form is known as hesperetin. Hesperidin is major flavone found in orange fruits. Hypotensive effect of hesperidin in acute and chronic glaucoma rats, glutamate level in vitreous humour and glutathione (GSH) level in aqueous humour were determined following 25, 50 and 100 mg/kg of hesperidin treatment. Acetazolamide (5 mg/kg) was used as positive control. Hesperidin treatment significantly reduced the increased intraocular pressure (IOP) level in dextrose induced ocular hypertension than saline treated rats. The effect of hesperidin was comparable to the positive control acetazolamide. Similarly, hesperidin treatment significantly reduced the IOP level in prednisolone acetate induced ocular hypertension than saline treated rats. In the aqueous humour, hesperidin treatment increased the glutathione level 125%, 184.4% and 231.2% at 25, 50 and 100 mg/kg of hesperidin respectively. In the vitreous humour, hesperidin treatment reduced the glutamate level 9.9%, 13.2% and 25.3% at 25, 50 and 100 mg/kg of hesperidin respectively. Histopathological analysis of normal saline treated rats showed morphological alteration in ciliary bodies. However, rats treated with hesperidin showed the reduced level of morphological alteration in ciliary bodies. Taking all these data together, it is suggested that the hesperidin supplementation was effective against glaucoma in experimental rats.

## Introduction

Glaucoma is well-known clinical eye conditions that damage the optic nerve due to abnormal pressure conditions in eye (Kingman 2004). Glaucoma is one of the major factors for irreversible blindness around the world over the age above 60 (Gossman et al. 2016). Researchers have reported that the vascular dysregulation and increased intraocular pressure (IOP) are major causative factors for glaucoma (Kyei et al. 2015), and these factors induces initial injury in the retinal glial cells (Chong and Martin 2015). Researchers have reported that the oxidative damage and injured neurons produced glycine and glutamate that induces excitotoxicity (Pose-Utrilla et al. 2017). Laser and incisional surgery, and medicines are available options for the management of glaucoma (Schwartz and Budenz 2004). Even though, several therapeutic agents available for treating glaucoma, still around 10% people suffering from the blindness all around the world (Acton 2013).

Researchers have reported that the polyphenols are major research area in the two decades due to their antioxidant potential (Tsao 2010; Gabriele et al. 2017). Several pharmacological and protective effects of polyphenols against cancer, degenerative disease, osteoporosis and cardiovascular disease (Scalbert et al. 2005a, b). Hesperidin is well-known glycoside widely present in the citrus fruits, and its aglycone form is known as hesperetin (Man et al. 2019). Hesperidin is major flavanone found in orange fruits (Kobayashi et al. 2008). Researchers have reported that the various therapeutic and protective effects of hesperidin against cerebral thrombosis, hypertension, fatty liver, osteoporosis, hypercholesterolemia and asthma (Qian et al. 2016; Chiba et al. 2014; Ikemura et al. 2012; Wei et al. 2012). However, there are no detailed studies available on the protective effect of hesperidin against glaucoma. Thus, we carried out the anti-glaucoma potential of hesperidin in experimentally-induced glaucoma model of rats.

## Materials and methods

### Chemicals

Acetazolamide (A6011, Sigma-Aldrich, China) was used as positive control for glaucoma. Proparacaine hydrochloride (1571001, Merck, China) was used as local anaesthetic ophthalmic solution. Prednisolone acetate (1556008, Merck, China) was used to induce ocular hypertension.

### Rats

Male albino rats were obtained from the Animal House of Xuzhou Medical University Affiliated Hospital of Lianyungang, China. The rats (170–200 g) were maintained in rat ages with standard atmospheric conditions of 12 h light and dark periods at 25 ± 0.5 °C with a relative humidity of 60 ± 5%. All methods were carried out in accordance with relevant guidelines and regulations, and approved by ethical committee of Xuzhou Medical University Affiliated Hospital of Lianyungang, China.

### Determination of hypotensive effect of hesperidin in acute glaucoma rats

The basal IOP levels in the eyes of rat were measured according to previously described method (Pang et al. 2005). A proper care was given to prevent nictitating membrane from arising under tonometer. The weights 5.5 g and 10 g was used to induce tension, and recorded. The mean value of both recordings was determined. Then, rats were grouped into normal saline (group I), acetazolamide (5 mg/kg, group II), 25 mg/kg of hesperidin (group III), 50 mg/kg of hesperidin (group IV) and 100 mg/kg of hesperidin (group V). All the doses were given to rats through oral gavage, and dose volume adjusted to 1 ml. Following 30 min of dose treatment, rats were administered dextrose solution (5%; 15 ml/kg) via marginal ear vein. The IOP value was measured for 2 h in every 20 min.

### Determination of ocular hypotensive effect of hesperidin in chronic glaucoma rats

#### Experimental model of ocular hypertension

The ocular hypertension was induced in rats through instilling prednisolone acetate (1%) in the eyes following baseline IOP determination two times per day, and it was continued for 21 days. The IOP level was measured in weekly basis between 8.30 and 9.00 AM. Symptoms such as fixed dilated pupils, limbal injection, bulging eyeball and limbal injection were observed in the rats having at least 50% increase in IOP (Dey et al. 2018).

### Determination of ocular hypertension effect of hesperidin

Rats with ocular hypertension were grouped into normal saline (group I), acetazolamide (5 mg/kg, group II), 25 mg/kg of hesperidin (group III), 50 mg/kg of hesperidin (group IV) and 100 mg/kg of hesperidin (group V). All the doses were given to rats through oral gavage, and dose volume adjusted to 1 ml. The dose given to rats for 21 consecutive days, and intraocular pressure was determined in all the eyes for every day (Dey et al. 2018).

### Determination of glutamate

In the vitreous humour, glutamate level was determined by using commercial kit (ab83389, glutamate assay kit, Abcam, UK). Briefly, vitreous humour was collected in the sterile tube and vitreous bodies were sonicated by using perchloric acid (0.2 M). Then, the homogenate was collected and centrifuged at 15,000*g* at cold temperature for 5 min, and then supernatant was collected for the determination of glutamate level. The standards and samples were prepared according to the instruction given in kit, and pipetted in a 96-well plate (Gao et al. 2018). The final absorbance was read at 405 nm in UV–VIS Spectrophotometer (Shimatzu, Guangdong, China).

### Determination of glutathione

In aqueous humour, the total glutathione was determined according to the instruction given in commercial kit (CS0260, glutathione assay kit, Merck, China). Anterior chamber was punctured by using 30-gauge needle after euthanizing the rats. Then, aqueous humour from all the eyes were collected in sterile tube. The triethanolamine (4 M) and metaphosphoric acid was used for the deproteining the aqueous humour. Finally, standards and aqueous humour samples were prepared according to the instruction given in kit, and pipetted in a 96-well plate, and incubated for 15 min at dark place (Iskusnykh et al. 2013). The final absorbance was read at 405 nm in UV–VIS Spectrophotometer (Shimadzu, Guangdong, China).

### Histopathological study

Enucleated eyes of rats were carefully removed in paraformaldehyde (10%), and then embedded in paraffin. Then, paraffin embedded eyes were sectioned and stained with haematoxylin and eosin. Then, stained sections were fixed in microscopic slides for the examination (Shibuya et al. 2015). The pathological examination was carried out by two independent pathologist at Xuzhou Medical University Affiliated Hospital of Lianyungang, China.

### Statistical analysis

Data are expressed as mean ± standard error of the mean (SEM). The groups were compared using Student’s *t*-tests and an analysis of variance (ANOVA). *P* values< 0.05 were considered to indicate statistical significance.

## Results

**Fig. 1:**
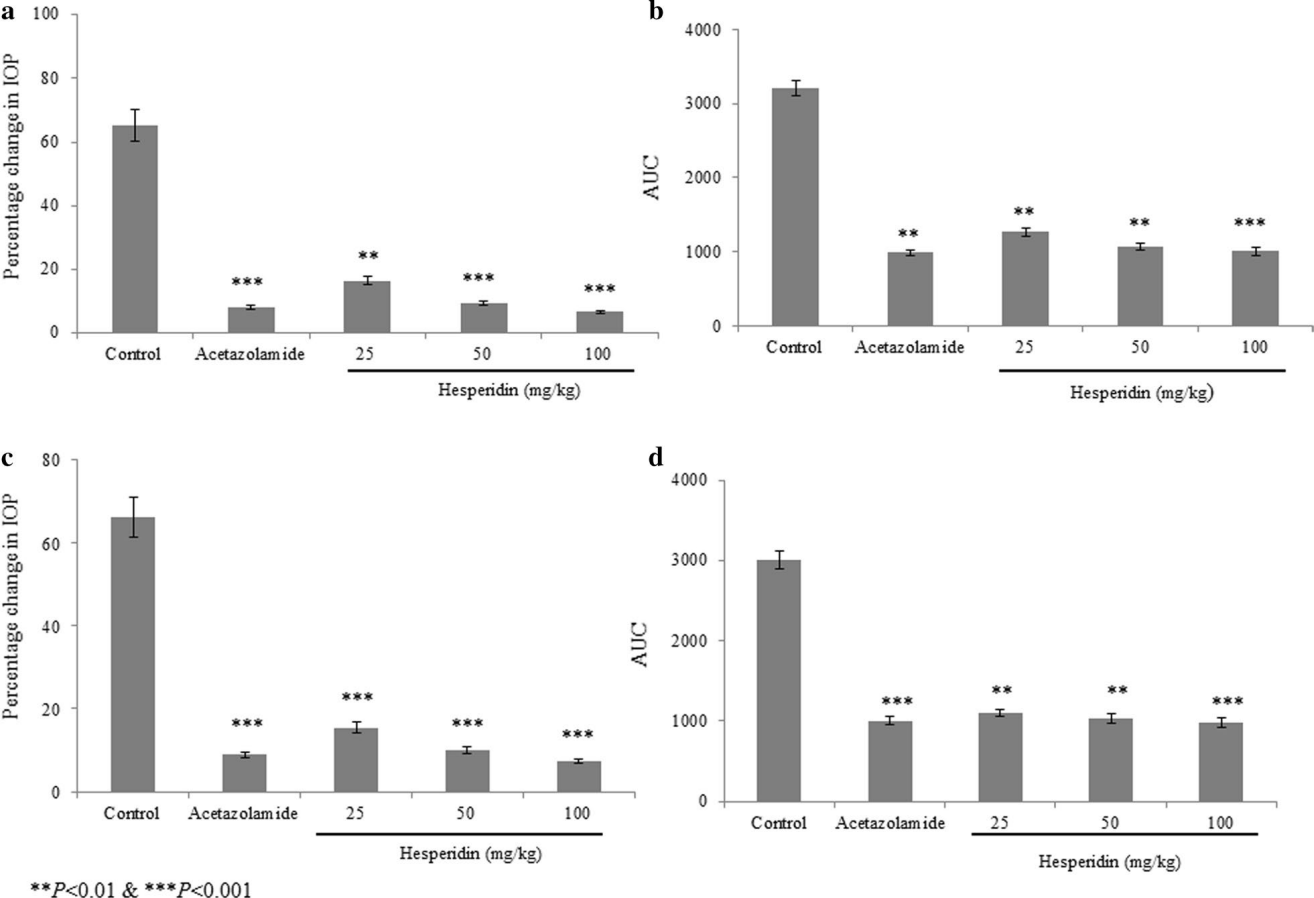
Anti-hypertensive effect of hesperidin in dextrose induced acute glaucoma rats. **a** and **b** represents the right eye. **c** and **d** represents the left eye. **P < 0.01 and ***P < 0.001 vs. group I. N = 6

**Fig. 2:**
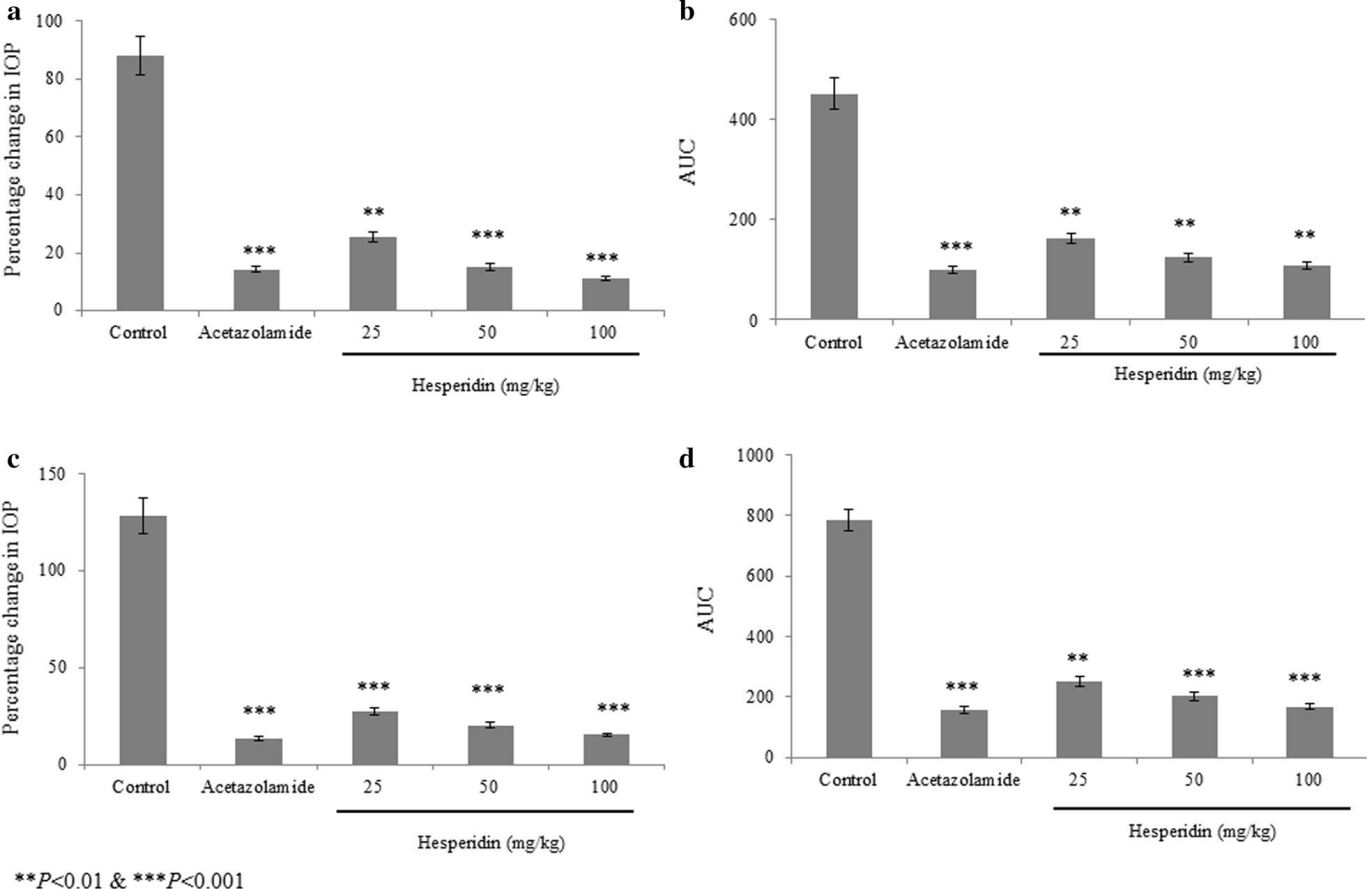
Anti-hypertensive effect of hesperidin in prednisolone acetate induced chronic glaucoma rats. **a** and **b** represents the right eye. **c** and **d** represents the left eye. **P < 0.01 and ***P < 0.001 vs. group I. N = 6

Hesperidin treatment significantly reduced the IOP level in dextrose induced ocular hypertension than saline treated rats (group I, P < 0.05; Fig. 1). The effect of hesperidin was comparable to the positive control acetazolamide, and there was no significant difference observed between hesperidin and acetazolamide (Fig. 1). Similarly, hesperidin treatment significantly reduced the IOP level in prednisolone acetate induced ocular hypertension than saline treated rats (group I, P < 0.05; Fig. 2). The effect of hesperidin was comparable to the positive control acetazolamide, and there was no significant difference observed between hesperidin and acetazolamide (Fig. 1).

**Fig. 3:**
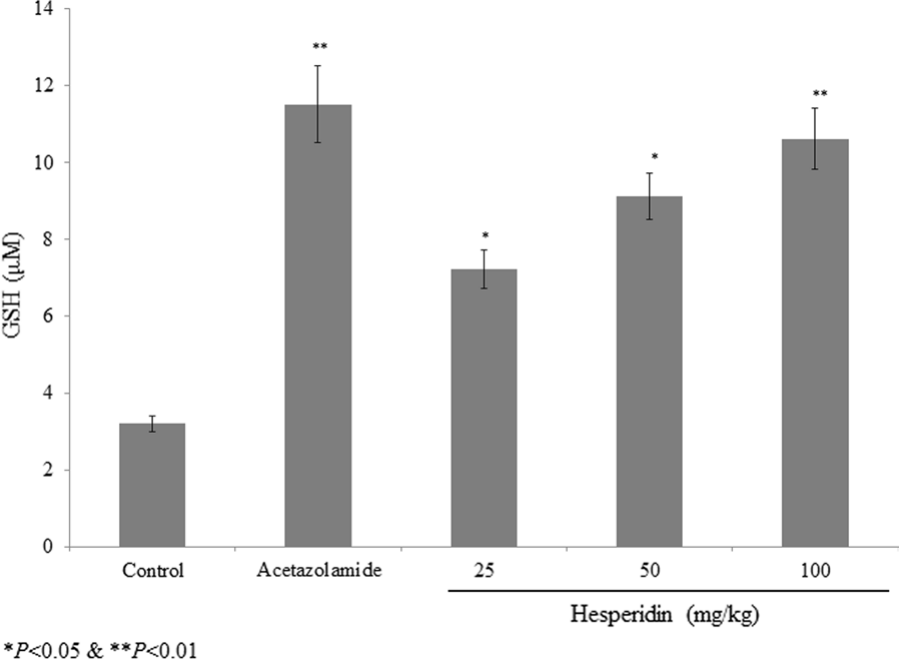
Protective effect of hesperidin on glutathione level in prednisolone acetate induced chronic glaucoma rats. **P < 0.01 and ***P < 0.001 vs. group I. N = 6

**Fig. 4:**
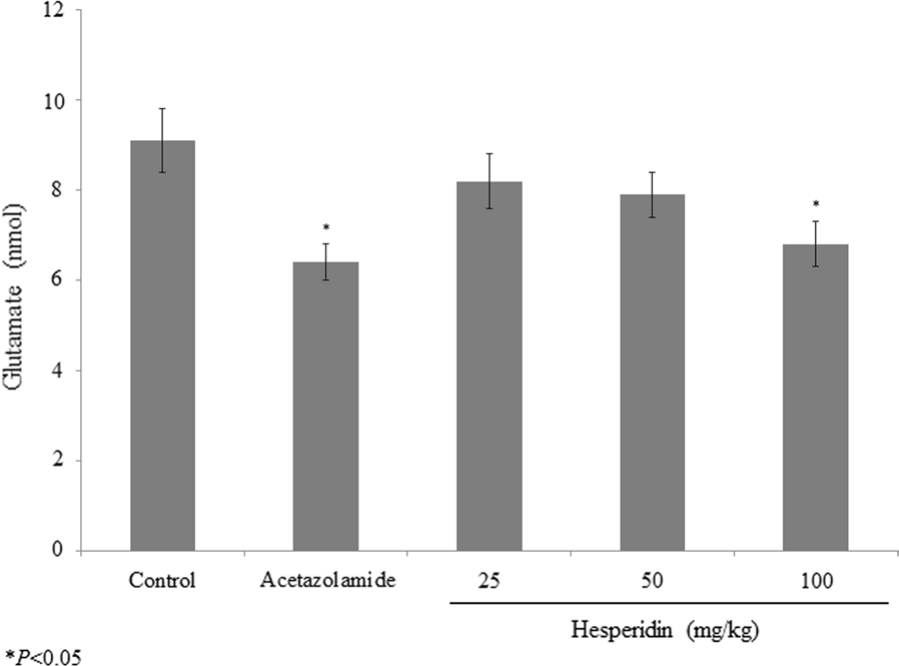
Protective effect of hesperidin on glutamate level in prednisolone acetate induced chronic glaucoma rats. **P < 0.01 and ***P < 0.001 vs. group I. N = 6

Hesperidin treatment prevented the oxidative stress through the recovery of glutathione content. In the aqueous humour, hesperidin treatment increased the glutathione level 125%, 184.4% and 231.2% at 25, 50 and 100 mg/kg of hesperidin respectively (P < 0.05; Fig. 3). Acetazolamide treatment increased the glutathione level 259.3% (P < 0.05; Fig. 3). In the vitreous humour, hesperidin treatment reduced the glutamate level 9.9%, 13.2% and 25.3% at 25, 50 and 100 mg/kg of hesperidin respectively (P < 0.05; Fig. 4). Acetazolamide treatment reduced the glutamate level 25.3% (P < 0.05; Fig. 4).

**Fig. 5:**
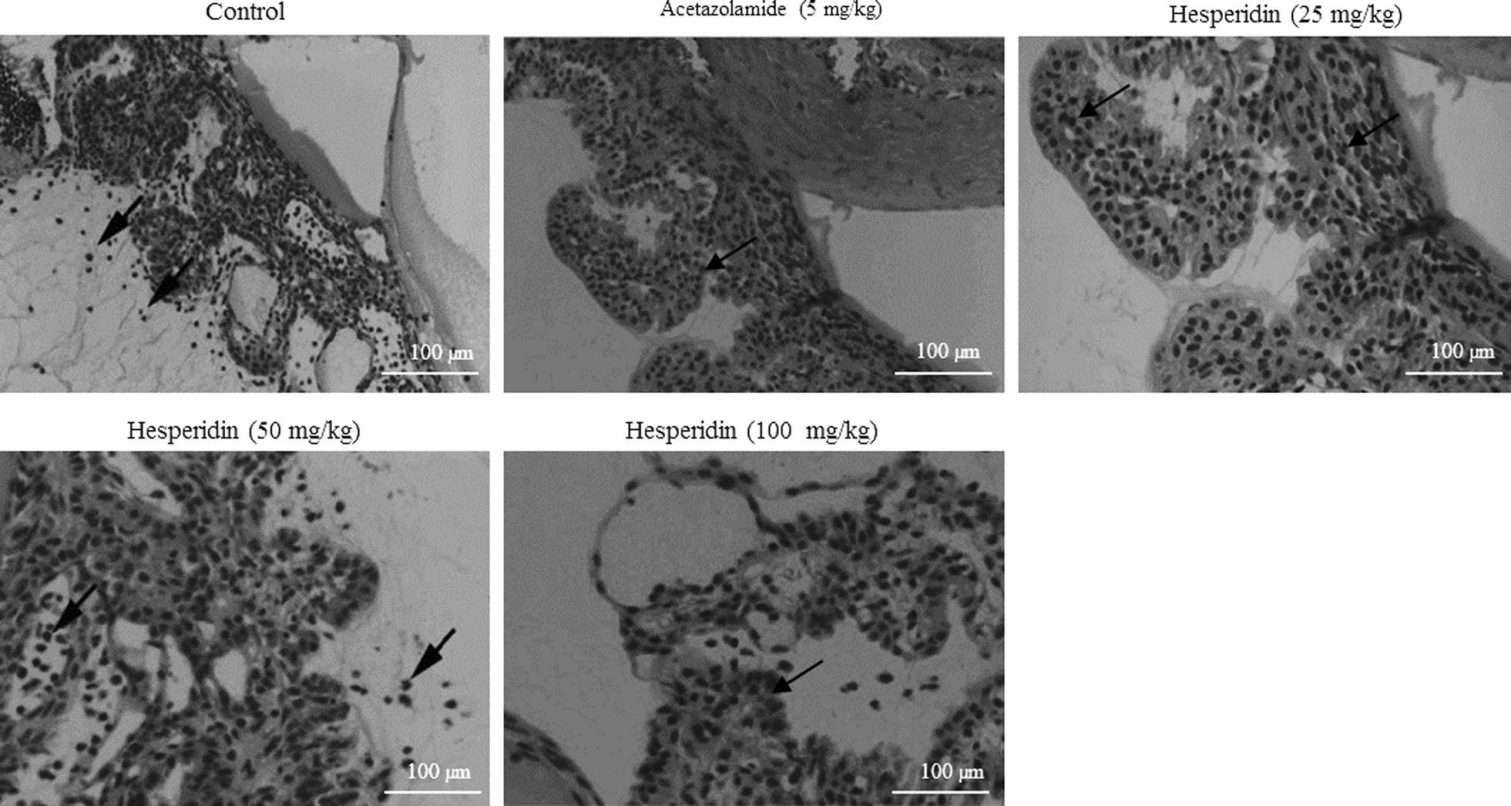
Histopathological analysis of anterior chamber in prednisolone acetate induced chronic glaucoma rats. N = 6. Scale bar is 100 µm

Histopathological analysis of normal saline treated rats showed morphological alteration in ciliary bodies. However, rats treated with hesperidin showed the reduced level of morphological alteration in ciliary bodies (Fig. 5). Acetazolamide treatment also showed the reduced level of morphological alteration in ciliary bodies (Fig. 5).

## Discussion

Glaucoma is well-known clinical eye conditions that damage the optic nerve due to abnormal pressure conditions in eye (Kingman 2004). Glaucoma is one of the major factors for irreversible blindness around the world over the age above 60 (Gossman et al. 2016). Researchers have reported that the vascular dysregulation and IOP are major causative factors for glaucoma (Kyei et al. 2015), and these factors induces initial injury in the retinal glial cells (Chong and Martin 2015). Researchers have reported that the oxidative damage and injured neurons produced glycine and glutamate that induces excitotoxicity (Pose-Utrilla et al. 2017). Laser and incisional surgery, and medicines are available options for the management of glaucoma (Schwartz and Budenz 2004). Even though, several therapeutic agents available for treating glaucoma, still around 10% people suffering from the blindness all around the world (Acton 2013).

Flavonoids are potential supplements for animal studies due to their lower toxicity at higher doses and prolonged period of treatment duration. In this study, we selected hesperidin due to their strong antioxidant potential. Researchers have reported that the various therapeutic and protective effects of hesperidin against cerebral thrombosis, hypertension, fatty liver, osteoporosis, hypercholesterolemia and asthma (Qian et al. 2016; Chiba et al. 2014; Ikemura et al. 2012; Wei et al. 2012). Menze et al. (2012) have reported the antioxidant activity of hesperidin like vitamin. Meloni (1995) have reported anti-inflammatory activity of hesperidin.

Kara et al. (2014) have reported the therapeutic effect of hesperetin against apoptosis in ischemia/reperfusion-induced retinal injury model of rats. Maekawa et al. (2017) have reported the neuroprotective effect of hesperidin in NMDA-induced retinal injury. Therapeutic potential of bioflavonoids against ocular disorders have been reported in several research (Majumdar and Srirangam 2010). Estruel-Amades et al. (2019) have reported the therapeutic potential of hesperidin against oxidative stress in rats. Anti-glaucoma effect of hesperidin was clearly established by evaluating the ocular hypotensive effect in chronic ocular hypertensive model. Researchers have reported the frequent intake of steroids induces oxidative stress, which leads to aqueous humour antioxidant system and cell loss of apoptotic trabecular meshwork. This, further leads to mitochondrial damage and mitochondrial dysfunction (Saccà et al. 2015). Saccà et al. (2011) have reported the reduction of aqueous production leads to reduced oxidative damage in trabecular meshwork and active mitochondria. Taking all these data together, it is suggested that the hesperidin supplementation was effective against glaucoma in experimental rats.

## Data Availability

Corresponding author could provide the all experimental data on valid request.

## Abbreviations

IOP: Increased intraocular pressure
GSH: Glutathione
SEM: Standard error of the mean
ANOVA: Analysis of variance
